# Inhibition of PFKFB3 Hampers the Progression of Atherosclerosis and Promotes Plaque Stability

**DOI:** 10.3389/fcell.2020.581641

**Published:** 2020-11-12

**Authors:** Kikkie Poels, Johan G. Schnitzler, Farahnaz Waissi, Johannes H. M. Levels, Erik S. G. Stroes, Mat J. A. P. Daemen, Esther Lutgens, Anne-Marije Pennekamp, Dominique P. V. De Kleijn, Tom T. P. Seijkens, Jeffrey Kroon

**Affiliations:** ^1^Department of Medical Biochemistry, Amsterdam Cardiovascular Sciences, Amsterdam University Medical Centers, University of Amsterdam, Amsterdam, Netherlands; ^2^Department of Experimental Vascular Medicine, Amsterdam University Medical Centers, University of Amsterdam, Amsterdam Cardiovascular Sciences, Amsterdam, Netherlands; ^3^Division of Surgical Specialties, Department of Vascular Surgery, University Medical Center Utrecht, Utrecht University, Utrecht, Netherlands; ^4^Netherlands Heart Institute, Utrecht, Netherlands; ^5^Department of Cardiology Amsterdam Cardiovascular Sciences, Amsterdam University Medical Centers, University of Amsterdam, Amsterdam, Netherlands; ^6^Department of Pathology, Amsterdam Cardiovascular Sciences (ACS), Amsterdam University Medical Centers, University of Amsterdam, Amsterdam, Netherlands; ^7^Institute for Cardiovascular Prevention (IPEK), Ludwig Maximilians University, Munich, Germany; ^8^German Center for Cardiovascular Research (DZHK), Partner Site Munich Heart Alliance, Munich, Germany; ^9^Department of Vascular Surgery, Netherlands and Netherlands Heart Institute, University Medical Center Utrecht, University Utrecht, Utrecht, Netherlands

**Keywords:** atherosclerosis, glycolysis, glycolytic inhibition, inflammation, plaque stability

## Abstract

**Aims:**

6-phosphofructo-2-kinase/fructose-2,6-biphosphatase (PFKFB)3-mediated glycolysis is pivotal in driving macrophage- and endothelial cell activation and thereby inflammation. Once activated, these cells play a crucial role in the progression of atherosclerosis. Here, we analyzed the expression of PFKFB3 in human atherosclerotic lesions and investigated the therapeutic potential of pharmacological inhibition of PFKFB3 in experimental atherosclerosis by using the glycolytic inhibitor PFK158.

**Methods and Results:**

PFKFB3 expression was higher in vulnerable human atheromatous carotid plaques when compared to stable fibrous plaques and predominantly expressed in plaque macrophages and endothelial cells. Analysis of advanced plaques of human coronary arteries revealed a positive correlation of PFKFB3 expression with necrotic core area. To further investigate the role of PFKFB3 in atherosclerotic disease progression, we treated 6–8 weeks old male *Ldlr*^–/–^ mice. These mice were fed a high cholesterol diet for 13 weeks, of which they were treated for 5 weeks with the glycolytic inhibitor PFK158 to block PFKFB3 activity. The incidence of fibrous cap atheroma (advanced plaques) was reduced in PFK158-treated mice. Plaque phenotype altered markedly as both necrotic core area and intraplaque apoptosis decreased. This coincided with thickening of the fibrous cap and increased plaque stability after PFK158 treatment. Concomitantly, we observed a decrease in glycolysis in peripheral blood mononuclear cells compared to the untreated group, which alludes that changes in the intracellular metabolism of monocyte and macrophages is advantageous for plaque stabilization.

**Conclusion:**

High PFKFB3 expression is associated with vulnerable atheromatous human carotid and coronary plaques. In mice, high PFKFB3 expression is also associated with a vulnerable plaque phenotype, whereas inhibition of PFKFB3 activity leads to plaque stabilization. This data implies that inhibition of inducible glycolysis may reduce inflammation, which has the ability to subsequently attenuate atherogenesis.

## Introduction

In recent years it has become clear that the development of atherosclerosis coincides with marked metabolic cellular alterations (Ali et al., 2018; Riksen and Stienstra, 2018). Particularly, inflammatory stimuli modify the intracellular metabolism of multiple cell types involved in atherogenesis, such as macrophages and endothelial cells. These cells are highly dependent on glycolysis for their energy metabolism to regulate cellular function (Bekkering et al., 2018; Riksen and Stienstra, 2018; Schnitzler et al., 2020). This induction in glycolysis could already be observed at regions where the endothelium is subjected to disturbed shear stress, making these early lesions susceptible for endothelial activation by lipoproteins, such as low-density lipoprotein (LDL) and lipoprotein(a) [Lp(a)] (Libby, 2012; Schnitzler et al., 2020). Lipid-lowering strategies markedly reduce cardiovascular event rates; however, a residual inflammatory risk remains even after potent lipid-lowering therapy in patients (Ridker, 2016; Stiekema et al., 2019).

Atherogenic stimuli such as Lp(a) have been shown to induce endothelial cell activation through upregulation of key-glycolytic players comprising glucose transporter (GLUT) 1 and hexokinase (Hk) II (Schnitzler et al., 2020). This glycolytic switch relies predominantly on the enzyme 6-phosphofructo-2-kinase/fructose-2,6-biphosphatase (PFKFB3), which serves as a potent source for inducible glycolysis (De Bock et al., 2013; Cantelmo et al., 2016). Conversely, inhibition of PFKFB3 leads to a marked reduction in the lipoprotein-induced inflammatory signature of endothelial cells and immune cells *in vitro*, pointing to a key role for PFKFB3 in the link between glycolysis and inflammation (Tawakol et al., 2015; Schnitzler et al., 2020). In addition, partial glycolytic inhibition in *Apoe*^–/–^ mice via silencing of PFKFB3 results in decreased glycolysis in the arterial wall (Tawakol et al., 2015). However, it is currently not known how glycolytic inhibition affects the progression of atherosclerosis.

In the present study, we analyzed the expression of PFKFB3 in human atherosclerotic lesions and explored the therapeutic potential of pharmacological inhibition of PFKFB3 in experimental atherosclerosis.

## Materials and Methods

### Immunohistochemical Analysis of Human Coronary Plaques

Human coronary artery specimens were obtained after informed written consent of the subjects during autopsies at the Department of Pathology of the Amsterdam University Medical Center (Amsterdam, Netherlands) and immediately fixed in 10% formalin and processed for paraffin embedding. The use of tissue was in agreement with the “Code for Proper Secondary Use of Human Tissue in Netherlands” and was in accordance with the principles as outlined in the Declaration of Helsinki. Based on fibrous cap formation and necrotic core size, specimens were classified as initial or advanced lesion, as described previously (Seijkens et al., 2019). PFKFB3 expression was analyzed by immunohistochemistry. After deparaffinization, slides were blocked for endogenous peroxidase activity in methanol, after which heat induced antigen retrieval was performed. Slides were then covered with 1:100 dilution of rabbit anti-human PFKFB3 antibody (Abcam, AB2617178) for an hour at room temperature. After washing with Tris-Hcl buffered saline (TBS), a secondary goat anti-rabbit biotin labeled antibody (Dako, E0432) was introduced 1:200 to the slides for 30 min, followed by 30 min of incubation with 1:200 ABC-HRP (for DAB) or ABC-AP kit (for Vector blue) (Vector, PK-6100, AK-5000). PFKFB3 was visualized with DAB (Vector SK-4105). For the double stainings, PFKFB3 was visualized with Vector blue (Vector, SK-5300), which was followed by 4% FCS block for 30 min and additional heat induced epitope retrieval. CD68 (mouse anti-human, Abcam, ab201340, 1:100), CD3 (rat anti-human, AbD Serotec, MCA1477, 1:100), αSMA (mouse anti-human, Sigma-Aldrich, F3777, 1:5,000) and CD31 (mouse anti-human, Novus Biologicals, NBP2-15202, 1:100) were used, all for 1 h at RT. Secondary goat anti-mouse biotinylated antibody (Dako, E0433) and rabbit anti-rat biotinylated antibody (Vector, BA-4001) were used at 1:300 for 30 min. Slides were covered in 1:200 ABC-HRP kit (Vector laboratories) for 30 min. Epitopes were visualized with ImmPACT AMEC red (Vector, SK-4285). Analyses of the slides was performed on a Leica DM3000 microscope with a DFC295 camera and further analysis was done with Adobe Photoshop CS6, Image J, and Las 4.1 software (Leica).

### Carotid Endarterectomy Specimens From the Athero-Express Biobank

The Athero-Express Biobank study design and plaque processing has been reported previously (Verhoeven et al., 2004,2005). In short, the plaques were randomly selected from patients undergoing a carotid endarterectomy. Percentage of atheroma was estimated by means of visual estimation using Picrosirius red with polarized light in combination with hematoxylin stains. Three groups were considered (*n* = 10 per group), based on the percentage of atheroma in the plaque being present: fibrous plaques containing < 10% fat; fibro-atheromatous, 10–40%; or atheromatous, > 40% fat. Next, the cumulative score for plaque vulnerability was determined by scoring macrophage-, collagen-, smooth muscle cell content and intraplaque hemorrhage. This plaque vulnerability index is scored as follows: macrophages: no/minor (0 points), moderate/heavy (1 point); collagen: moderate/heavy (0 points), no/minor (1 point); smooth muscle cells: moderate/heavy (0 points), no/minor (1 point). Intraplaque hemorrhage was scored as absent (0 points) or present (1 point), as previously described (Hellings et al., 2010). Sections were subsequently stained as described previously (Schnitzler et al., 2020). Briefly, plaque sections were dewaxed in xylene and dehydrated in graduated concentration of ethanol (100–70% ethanol). Heat-induced epitope retrieval was performed before blocking the sections for 20 min with 5% BSA in tris-buffered saline (TBS). Next, the sections were incubated for 2 h at room temperature with the following primary antibodies: PFKFB3 rabbit anti-human (Abcam, AB2617178), CD68 (Invitrogen, MA1-80133) and von Willebrand Factor mouse anti-human (Agilent, AB2216702). Next, sections were incubated in the dark at RT with biotinylated secondary antibody: goat anti-rabbit Alexa 488 (Thermo Fisher Scientific, AB 143165), goat anti-mouse Alexa 564 (Thermo Fisher Scientific, AB2536161) and goat anti-mouse Alexa 647 (Thermo Fisher Scientific, AB 2535804). After 1 h incubation, the sections were mounted with DAKO mounting medium containing DAPI (Agilent). A Leica TCS SP8 Confocal laser scanning microscope was used to image the sections and quantification was performed using Leica LAS-X software (Leica Camera, Wetzlar, Germany). All patients provided written informed consent. The Athero-Express study protocol conforms to the Declaration of Helsinki and has been approved by the Institution’s ethics committee on research on humans.

### Animal Experiments

Male *Ldlr*^–/–^ mice were bred and housed at the local animal facility with normal light/dark cycle and were fed 0.15% cholesterol diet *ad libitum*. At 16 weeks of age mice were injected in the morning 3x/week for 5 weeks with PFK158 (2 mg/kg *ip*) or vehicle control (0.1% DMSO). Mice were euthanized by a single intraperitoneal injection of a cocktail of 30 mg/kg Sedamun (xylazine 20 mg/ml) and 100 mg/kg Anesketin (ketamine, 100 mg/ml) and death was confirmed by heart puncture. All experiments were approved by the Committee for Animal Welfare of the University of Amsterdam, Netherlands (protocol 265-AW-1), and comply to the European Regulations as identified in Directive 2010/63/EU on the protection of laboratory animals.

### Lipoprotein Separation by Fast Protein Liquid Chromatography

Blood was obtained by venous or cardiac puncture and collected into ethylenediaminetetraacetic acid (EDTA)-containing tubes. Individual lipoprotein levels were determined by fast-performance liquid chromatography (FPLC) as described previously (Fedoseienko et al., 2018). In short, total cholesterol (TC) and triglyceride (TG) content in the main lipoprotein classes (VLDL, LDL, and HDL) was determined using FPLC. The main system consisted of a PU-980 ternary pump with an LG-980-02 linear degasser and an UV-975 UV/VIS detector (Jasco, Tokyo, Japan). After injection of 30 μl plasma (1:1 diluted with TBS) the lipoproteins were separated using a Superose 6 increase 10/30 column (GE Healthcare Hoevelaken, Netherlands). As eluent TBS pH 7.4 was used at a flow rate of 0.31 ml/min. A second pump (PU-2080i Plus, Jasco, Tokyo Japan) was used for either in-line cholesterol PAP or Triglyceride enzymatic substrate reagent (Sopachem, Ochten, Netherlands) addition at a flowrate of 0.1 ml/min facilitating TC or TC detection. Commercially available lipid plasma standards (low, medium and high) were used for generation of TC or TG calibration curves for the quantitative analysis (SKZL, Nijmegen, Netherlands) of the separated lipoprotein fractions. All calculations performed on the chromatograms were carried out with ChromNav chromatographic software, version 1.0 (Jasco, Tokyo, Japan).

### Histology

At the age of 21 weeks, mice were sacrificed and the arterial tree was perfused with PBS and 1% paraformaldehyde. The aortic arch and organs were isolated and fixed in paraformaldehyde. Longitudinal sections of the aortic arch (4 μm) (*n* = 10/group) and sections of the three valve area in the aortic root (*n* = 14/group) were stained with hematoxylin and eosin and analyzed for plaque extent and phenotype as previously described (Seijkens et al., 2018). One mouse was excluded from aortic arch analysis due to situs inversus. Necrotic core measurements were only performed in advanced plaques. Intimal xanthoma (IX) was defined by a small lesion consisting of foam cells in which no extracellular lipid accumulation can be detected, pathological intimal thickening (PIT) was defined as a larger lesion that mainly consists of macrophage foam cells, but contains small extracellular lipid pools and the first matrix depositions, a fibrous cap atheroma (FCA) was defined as an advanced atherosclerotic lesion with a clear fibrous cap and necrotic cores (extracellular lipid accumulation, cholesterol crystals and/or calcification; Virmani et al., 2000). Fibrous cap thickness was measured at the thinnest part of each fibrous cap. Immunohistochemistry was performed for MAC3 (BD Pharmingen), alpha smooth muscle actin (Sigma-Aldrich), CD3 (AbD Serotec), CD8 (eBioscience), Ki67 (Abcam) and TUNEL (*in situ* apoptosis detection kit, Roche). The stability index was defined as (% α smooth muscle actin/% necrotic core; Poels et al., 2020). Double immunohistochemical stainings were performed with rabbit anti-mouse PFKFB3 antibody (Abcam, AB2617178) in combination with CD31 (Dianova, Hamburg, Germany), and the previously mentioned MAC3, CD3 and alpha smooth muscle actin. Morphometric analyses were performed on a Leica DM3000 microscope with a DFC 295 camera and Adobe Photoshop CS6, Image J or Las4.0 software (Leica).

### Flow Cytometry

Spleen and lymph nodes were homogenized, and blood and spleen samples were subjected to red blood cell lysis. The cells were stained with fluorescently labeled surface antibodies (CD45, CD3, CD4, CD8, F4/80, Ly6C, Ly6G, MHCI, MHCII, CD44, CD62L, CD19, CD11C, NK1.1; all from BD Biosciences). For intracellular staining, cells were fixed and permeabilized with fixation/permeabilization buffer (eBioscience) and stained with fluorescent antibodies against Foxp3 (BioLegend). Flow cytometric analysis was performed on a BD Canto II (BD Biosciences).

### Immunofluorescent Staining of Murine Aortas Using Confocal Microscopy

Abdominal aortas remained intact for *en face* staining with ICAM-1 (Abcam) and VCAM-1 (Abcam) or aortas were dissected until 3–5 mm before the iliac bifurcation to obtain transverse sections. For the latter, aortas were embedded in paraffin for subsequent cutting sections into 10 μm slices for immunofluorescent staining. Next, the transverse sections were stained as described previously (Schnitzler et al., 2020). In summary, the following antibodies were used: ICAM-1 (Abcam), GLUT-1 (Thermo Fisher) and secondary antibody Alexa Fluor 568 for PFKFB3 and Alexa 633 for GLUT1-1 (both Thermo Fisher). HIF1α (R&D systems, MAB1536), GLUT3 (Invitrogen, MA5-32697), and secondary Alexa Fluor 647 for HIF1a and Alexa Fluor 488 for GLUT3 (both Thermo Fisher Scientific). The transverse sections aortas were fixed with DAKO mounting medium containing DAPI (Agilent, Santa Clara, United States). All samples were visualized on a Leica TCS SP8 Confocal laser scanning microscope and quantified using ImageJ.

### RNA Isolation, cDNA Synthesis and Real Time Quantitative Polymerase Chain Reaction

Tissues were homogenized and lysed with TriPure (Roche, Basel, Switzerland). RNA was isolated according to the manufacturer’s instructions. Briefly, 1 μg of RNA was used for cDNA synthesis with iScript (BioRad, Veenendaal, Netherlands). qPCR was performed using Sybr Green Fast (Bioline Meridian Bioscience, Cincinnati, OH, United States) on a ViiA7 PCR machine (Applied Biosystems, Bleiswijk, Netherlands). Gene expression was normalized to the housekeeping gene *H36B4*. Primer sequences are shown in Supplementary Table 1. All gene expression graphs indicate fold change of relative gene expression of which values were normalized to the mean of the control group.

### Murine PBMC Isolation and Seahorse Flux Analysis

Murine peripheral blood mononuclear cells (PBMCs) were obtained from whole blood samples through density centrifugation using Lymphoprep (Stemcell Technologies, Koln, Germany; *D* = 1.077 g/mL) as described in detail previously (Schnitzler et al., 2017). In short, blood was diluted in a 1:1 ratio with PBS enriched with 2 mM EDTA and subsequently added to a layer of Lymphoprep. Next, cells were centrifuged for 20 min at 600× g at RT with slow acceleration and no brake. The PBMC fraction was collected and washed twice with PBS/2 mM EDTA. Next, cells were counted using a Casy Counter (Roche Innovatis Casy TT, Bielefeld, Germany). Next, PBMCs were seeded in 80 μL EGM-2 medium at a density of 50,000 cells per well on XFe96 microplates (Seahorse Bioscience). The cells were incubated in unbuffered DMEM assay medium (Merck) for 1.5 h in a non-CO_2_ incubator at 37°C. A Seahorse XFe 96 analyzer (Seahorse Bioscience, Billerica, United States) was used to analyze cellular respiration. Extracellular acidification rates (ECAR) were measured after injecting glucose (10 mM), the mitochondrial/ATP synthase inhibitor oligomycin (1.5 μM), and the glycolysis inhibitor 2-Deoxy-D-glucose (2-DG; 100 mM), to determine glycolysis, glycolytic capacity and glycolytic reserve, respectively. Values were corrected for cell count.

### Quantification and Statistical Analysis

Data are presented as the mean ± SEM, unless stated otherwise. To avoid observer bias, the murine and human plaque analysis were blinded. Experiments were assessed with two-tailed Student’s *t*-tests, two-tailed Mann Whitney or One-way ANOVA as indicated in the figure legends. Clinical characteristics of patients from the Athero-Express Biobank were compared between the three plaque phenotypically different groups (fibrous, fibro-atheromatous, and atheromatous plaques). Statistical testing for differences between the three group was done by using a One-way ANOVA test for normally distributed data and a Kruskal–Wallis Rank Sum Test for non-normally distributed data [i.e., triglycerides and lipoprotein(a)]. Representative images were selected based on the value closest to the mean value per group. Statistics were performed using Graphpad Prism (v8.0h; La Jolla, CA). The analysis found in the baseline table was performed in R version 3.6.1 (R Foundation, Vienna, Austria). A *P*-value of < 0.05 was considered statistically significant.

## Results

### PFKFB3 Expression in Macrophages and Endothelial Cells Correlates With Plaque Instability in Human Atherosclerosis

**FIGURE 1:**
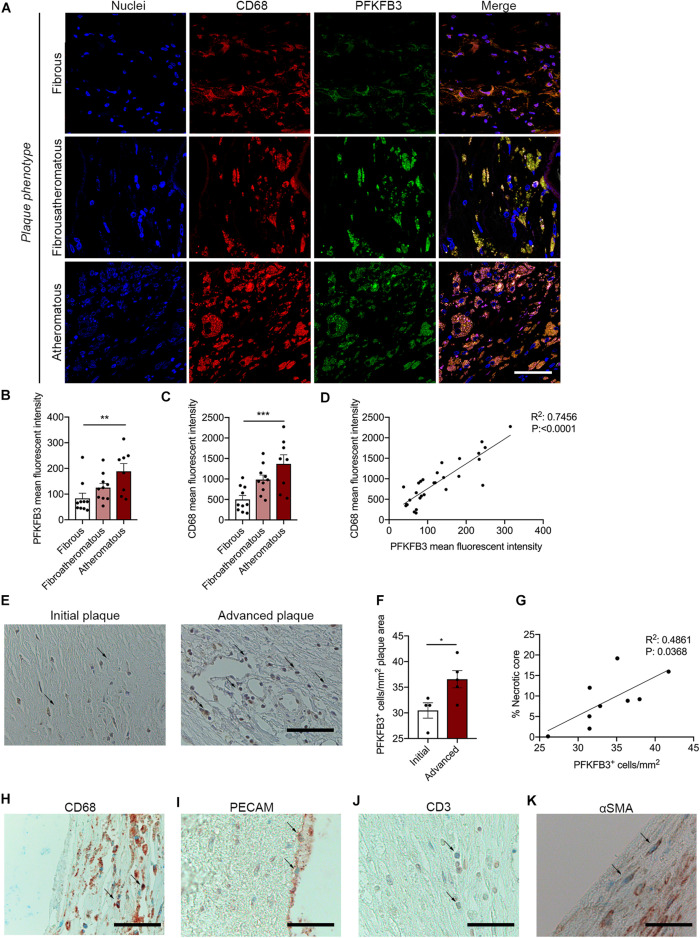
PFKFB3 expression is increased in advanced atherosclerotic plaques and correlates with CD68^+^ macrophages and necrotic core area. **(A,B)** Increased expression of PFKFB3 (green) in atheromatous plaques compared with fibrous carotid plaques. *n* = 10 for fibrous and fibroatheromatous plaques, *n* = 8 for atheromatous plaques; one-way ANOVA, *P* = 0.0078 for fibrous vs. atheromatous plaques. Scale bar 50 μm. **(A,C)** CD68^+^ macrophages (red) increased in atheromatous carotid plaques. *n* = 10 for fibrous and fibroatheromatous plaques, *n* = 8 for atheromatous plaques; one-way ANOVA, *P* = 0.0008 for fibrous vs. atheromatous plaques. Scale bar 50 μm **(D)** PFKFB3 expression and CD68 expression correlated significantly. *n* = 28; linear regression analysis, *R*^2^ = 0.7456, *P* < 0.0001. **(E)** Human coronary atherosclerotic advanced plaques showed increased PFKFB3 expression compared to initial plaques. *n* = 4 for initial vs. *n* = 5 for advanced plaques; two-tailed unpaired Mann-Whitney, *P* = 0.0317. Scale bar 200 μm. **(F)** PFKFB3 expression positively correlated with necrotic core area. *n* = 9; Linear regression analysis *R*^2^ = 0.4861, *P* = 0.0368. **(G)** Immunohistochemistry of PFKFB3^+^ co-staining with CD68^+^ macrophages; Scale bar 100 μm, **(H)** PECAM^+^ endothelial cells; Scale bar 100 μm, **(I)** CD3^+^ T cells; Scale bar 100 μm, and **(J)** and α smooth muscle actin (αSMA)^+^ vascular smooth muscle cells **(K)**; Scale bar 100 μm. Data are shown as mean ± standard error of the mean. *P < 0.05, **P ≤ 0.005, ***P < 0.0005. PFKFB3, 6-phophofructo-2-kinase/fructose-2,6-biphosphatase 3; PECAM, platelet endothelial cell adhesion molecule; αSMA.

Carotid plaques were obtained from patients undergoing endarterectomy enrolled in the Athero-Express biobank. Plaques were classified as fibrous plaques (containing < 10% intraplaque fat), fibro-atheromatous (10–40% intraplaque fat) or atheromatous (>40% intraplaque fat; Verhoeven et al., 2004, 2005). Both fibro-atheromatous and atheromatous plaques had an increased plaque vulnerability index (Table 1; Verhoeven et al., 2005). Further carotid plaque analysis revealed a marked higher PFKFB3 expression when plaque vulnerability is higher (Figures 1A,B). Higher PFKFB3 expression coincides with high number of CD68^+^ macrophage in atheromatous plaques (Figures 1A,C) that positively correlates with PFKFB3 expression (Figure 1D). These data imply that mainly macrophages show enhanced PFKFB3 expression in atherosclerotic plaques. In line, human coronary atherosclerotic plaques, histologically classified as fibrous cap atheromata (advanced atherosclerosis) express higher levels of PFKFB3^+^ cells when compared with intimal xanthomas or pathological intimal thickenings (initial/intermediate atherosclerosis) (Figures 1E,F). PFKFB3 expression positively correlates with necrotic core area (Figure 1G), indicating that PFKFB3 expression increases during the progression of atherosclerosis. The majority of PFKFB3^+^ cells are CD68^+^ macrophages and PECAM^+^ endothelial cells (Figures 1H,I). Only few CD3^+^ T cells and α smooth muscle actin (αSMA)^+^ vascular smooth muscle cells express PFKFB3 (Figures 1J,K). A similar expression is observed in murine plaques, where PFKFB3 was also predominantly observed in macrophages (MAC3^+^) and the endothelium (PECAM^+^) and to a lesser extend in T-cells (CD3^+^) and smooth muscle cells (αSMA^+^) (Supplementary Figures 1A–D). Together, these data suggest that PFKFB3 expression increases as plaques become more vulnerable.

**TABLE 1:** Clinical characteristics of included patients of Athero-Express Biobank.

	Fibrous (*n* = 10)	Fibro-Atheromatous (*n* = 10)	Atheromatous (*n* = 10)	*P*-value
Age	73.1 (7.5)	67.7 (8.7)	69.4 (7.4)	0.308
Gender (Male, %)	6 (60.0)	8 (80.0)	8 (80.0)	0.506
Systolic blood pressure, mmHg	148.3 (33.6)	174.7 (45.6)	176.7 (37.3)	0.316
BMI, kg/m^2^	26.8 (4.5)	26.0 (2.5)	27.2 (3.8)	0.768
Current smoker (yes, %)	2 (20.0)	4 (40.0)	4 (40.0)	0.549*
Cholesterol lowering medication (yes, %)	9 (90.0)	8 (80.0)	8 (80.0)	0.787
Total cholesterol, mmol/L	3.9 (0.7)	4.3 (0.9)	4.0 (1.3)	0.607
HDL-cholesterol, mmol/L	1.0 (0.3)	1.1 (0.3)	1.0 (0.2)	0.587
LDL-cholesterol, mmol/L	2.0 (0.3)	2.3 (1.0)	2.0 (1.1)	0.709
Triglycerides, mmol/L	1.7 [1.3, 2.5]	1.4 [1.0, 1.8]	1.3 [0.9, 1.8]	0.399
Lipoprotein(a), nmol/L	21.4 [11.1, 44.6]	49.4 [14.0, 121.5]	14.2 [12.2, 47.0]	0.464
Glucose, mmol/L	5.9 (2.1)	6.7 (3.5)	6.8 (3.0)	0.794
Plaque vulnerability index	0.0 (0.0)	1.4 (0.5)	3.8 (0.4)	<0.001

*Chi-Square test, 1-sided. Data are presented as mean (SD), n (%) or median [IQR]. BMI, body-mass index; HDL, high density lipoprotein; LDL, low-density lipoprotein.

### PFKFB3 Inhibition Hampers the Progression of Atherosclerosis and Promotes Plaque Stability

**FIGURE 2:**
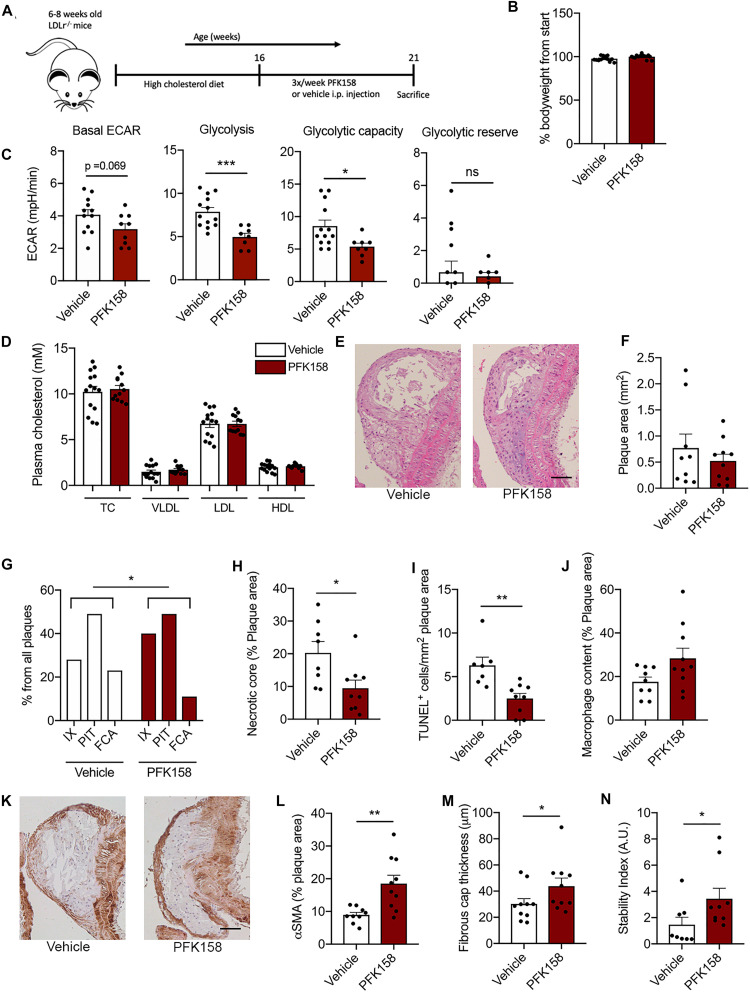
PFK158 hampers the progression of atherosclerosis and increases plaque stability. **(A)** Experimental design: after 8 weeks of 0.15% high cholesterol diet male *Ldlr*^–/–^ mice (*n* = 14/group) were injected 3 times per week with either PFK158 or vehicle for 5 weeks (13 weeks of high fat diet in total). **(B)** PFK158 treatment did not significantly affect bodyweight. **(C)** Analysis of circulating peripheral blood mononuclear cells revealed an increased extracellular acidification rate in vehicle cells (*n* = 13) compared to their PFK158-treated counterparts (*n* = 8) indicating the effectiveness of glycolytic inhibition. Two-tailed unpaired Student’s *t*-test *P* = 0.0007. *n* = 10/group. **(D)** Plasma cholesterol levels remained similar between groups. *n* = 14/group **(E,F)** Atherosclerotic lesion area in the aortic arch did not change; scale bar = 50 μm. *n* = 10/group. **(G)** Morphological analysis (Virmani classification) revealed a reduced incidence of FCA in the aortic arch of PFK158 treated mice. The incidence of intimal xanthomas was increased in PFK158-treated mice. Chi-square, *P* = 0.0417. **(H)** Necrotic core area per advanced plaque was reduced in PFK158 treated mice (*n* = 8) vs. vehicle (*n* = 9). Unpaired Student’s *t*-test *P* = 0.0205. **(I)** PFK158 treatment (*n* = 9) reduced the number of apoptotic (TUNEL^+^) cells compared to vehicle (*n* = 7). Two-tailed unpaired Mann-Whitney, *P* = 0.0012. **(J)** A trend toward increased macrophage content was observed in PFK158 treated mice. Two-tailed unpaired Student’s *t*-test *P* = 0.0628. **(K,L)** PFK158 increased αSMA content in PFK158 treated mice (*n* = 10) vs. vehicle treated mice (*n* = 9; Two-tailed unpaired Student’s *t*-test *P* = 0.0033; scale bar = 50 μm) and **(M)** increased fibrous cap thickness (*n* = 10/group; Two-tailed unpaired Mann-Whitney, *P* = 0.0410). **(N)** which increased the plaque stability index (*n* = 8 for vehicle and *n* = 9 for PFK158 group; Two-tailed unpaired Mann-Whitney, *P* = 0.0464). Data are shown as mean ± standard error of the mean. *P < 0.05, **P < 0.005, ***P < 0.0005. TC, total cholesterol; VLDL, Very low-density lipoprotein; LDL, low-density lipoprotein; HDL, high-density lipoprotein; H&E, hematoxylin and eosin; IX, initial xanthoma; PIT, pathologic intimal thickening; FCA, fibrous cap atheroma; αSMA, α smooth muscle actin; PBMC, peripheral blood mononuclear cell; ECAR, extracellular acidification rate.

To investigate the therapeutic potential of PFKFB3 inhibition on atherosclerosis, 6–8 weeks-old *Ldlr*^–/–^ mice received a high fat diet for 8 weeks and were subsequently treated with the PFKFB3 inhibitor PFK158 for 5 weeks (intraperitoneal administration, 3 times per week, 2 mg/kg) (Figure 2A). PFK158 treatment had no effect on bodyweight (Figure 2B). To assess the efficacy of PFK158, we examined the metabolic signature of peripheral blood mononuclear cells (PBMCs) of PFK158-treated mice which displayed a significant decrease in glycolysis and their glycolytic capacity (Figure 2C). PFK158-treatment had no effect on total plasma cholesterol levels, plasma VLDL-, LDL- and HDL-levels (Figure 2D). Immune cell distribution in peripheral blood and lymphoid organs was unaffected by PFK158 treatment (Supplementary Figures 1E–H).

While atherosclerotic lesion size in the aortic arch was not affected by PFK158 treatment (Figures 2E,F), morphological analysis demonstrated that the aortic arch of PFK158-treated mice contained relatively less FCA (advanced atherosclerotic lesions; Figure 2G). This is accompanied by an increase in the incidence of intimal xanthomas (initial atherosclerotic lesions) upon PFK158 treatment (Figure 2G), indicating that inhibition of PFKFB3 mitigates progression of atherosclerosis. Interestingly, both the necrotic core area per plaque (Figure 2H) and intraplaque apoptosis (Figure 2I) were reduced in PFK158-treated mice, whilst the number of CD4^+^ and CD8^+^ T cells and proliferating Ki67^+^ cells remained unaffected (Supplementary Figures 2A–C, respectively). In addition, a trend toward increased plaque macrophage content (Mac3^+^ area) was observed in PFK158 treated mice (Figure 2J). Interestingly, PFK158 treatment induced a marked increase in vascular smooth muscle content (αSMA^+^) (Figures 2K,L) and thickening of the fibrous cap (Figure 2M). Together this led to a significant rise in stable plaques as shown by an increased plaque stability index (Figure 2N). A similar atherosclerotic phenotype was observed in the aortic roots of PFK158 treated mice (Supplementary Figures 3A–F). These data indicate that therapeutic inhibition of PFKFB3 inhibits the progression of atherosclerosis and promotes plaque stability.

### PFK158 Does Not Affect the Atherosclerotic Phenotype of Endothelium

**FIGURE 3:**
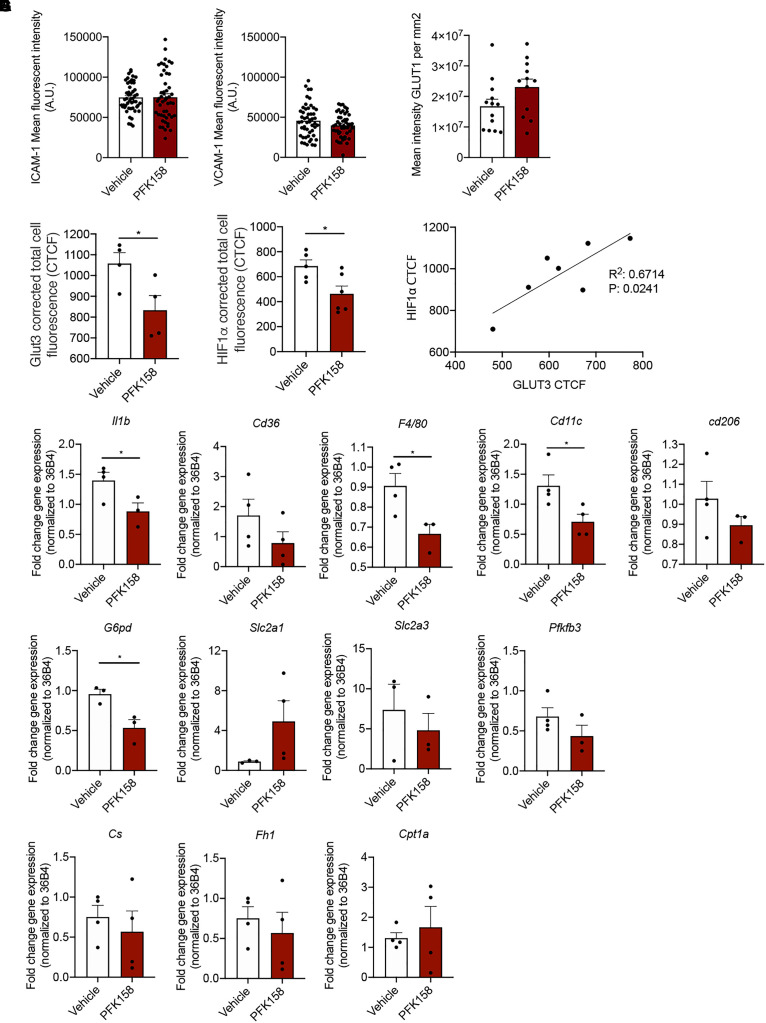
PFK158 alters phenotype of highly vascularized lung tissue but does not affect arterial endothelial phenotype. **(A)** ICAM-1 and VCAM-1 expression per image (*n* = 50 per group) and **(B)** GLUT1 expression in ECs did not significantly alter between groups (*n* = 13 for vehicle and *n* = 12 for PFK158 group). **(C)** GLUT3 expression was significantly decreased after PFK158-treatment (*n* = 4 for both groups) which coincided with decreased. **(D)** HIF1α levels as well (*n* = 5 for vehicle and *n* = 6 for PFK158 group). **(E)** A positive correlation (*R*^2^: 0.6714, *P*: 0.0241) was observed between HIF1α and GLUT3 expression levels. Homogenates of plaque-containing aortic arches indicated several significantly differentially expressed genes. **(F–H)** Two-tailed unpaired Student’s *t*-test, *P* = 0.0497 for *Il1*β, *P* = 0.0239 for *G6pd, P* = 0.0347 for *F4/80*, and *P* = 0.0348 for *Cd11c* (*n* = 3–4 per group). Data are shown as mean ± standard error of the mean. *P < 0.05. ICAM-1, intercellular adhesion molecule 1; GLUT1, glucose transporter 1, CTCF, corrected total cell fluorescence.

No differences in ICAM-1 and VCAM-1 expression were observed after PFK158 treatment (Figure 3A). Previously, we have shown that GLUT1 protein expression decreases in arterial ECs after PFK158 treatment (Schnitzler et al., 2020); however GLUT1 expression remained unaffected in cross-sections of aortas between groups (Figure 3B). Interestingly, after PFK158 treatment we observed a significant downregulation of both GLUT3 (Figure 3C) and HIF1α (Figure 3D) in the plaques. We furthermore observed a significant positive correlation between HIF1α and Glut3 expression levels (Figure 3E), suggesting that HIF1α and GLUT3 levels are regulated via PFKFB3-mediated glycolysis and that increased levels of HIF1α and GLUT3 are associated with increased plaque stability. Next, homogenates of murine plaque-containing aortic arches indicated that PFK158 treatment did result in a decrease in the gene expression of inflammatory markers *Il1*β and a trend in *Cd36*. Conversely, macrophage marker *F4/80* decreased in the PFK158 group, with a concomitant significant reduction in *Cd11c* and *Cd206*, suggesting a lower content of alternatively activated macrophages (Figure 3F). Interestingly, we also observed reduced *G6pd* transcript levels, the first and rate-limiting enzyme in the Pentose Phosphate Pathway (PPP), after PFK158 treatment, whereas *Slc2a1, Slc2a3* and the glycolytic mediator *Pfkfb3* were not altered by PFK158 treatment (Figure 3G). Similarly, *Cs, Fh1* involved in the TCA cycle, as well as *Cpt1a*, involved in fatty acid oxidation were not altered by the treatment (Figure 3H). In conclusion, these data are indicative of an altered inflammatory profile in the plaques of aortic arches on the level of gene expression after PFK158 treatment.

## Discussion

In this study, we reveal for the first time that PFKFB3 expression positively correlates with an unstable plaque phenotype in both carotid and coronary plaques in humans. Targeting PFKFB3 with a low-dose glycolytic inhibitor leads a reduction in advanced plaques with a vulnerable phenotype and an increase in stable plaque phenotype in *Ldlr*^–/–^ mice, with a concomitant decrease in glycolytic flux in circulating PBMCs. This data may imply an atheroprotective role for glycolytic inhibition.

In humans, increased plaque vulnerability is characterized by a profound inflammatory and glycolytic phenotype (Tomas et al., 2018) as shown by metabolomic analysis on 156 human carotid endarterectomy plaques. They reported that atherosclerotic plaques with a high risk to rupture are recognized by increased glycophorin A (indicative of intraplaque hemorrhage) and a profound glycolytic phenotype (Tomas et al., 2018). Here, we find a positive correlation between PFKFB3 expression and necrotic core area in human coronary plaques and a positive correlation between PFBFB3 and high-risk plaques, mainly attributable to high expression of PFKFB3 in macrophages in both human carotid and coronary plaques (Bekkering et al., 2016). In addition, the increasing vulnerability of carotid plaques is positively associated with an increased prevalence of transient ischemic attack and stroke (Verhoeven et al., 2005). We also substantiate a marked increase in CD68^+^ macrophage content in advanced carotid plaques (Moore et al., 2013; Tomas et al., 2018). Once macrophages are activated, they increase PFKFB3 activity, indicating that inflammation and glycolytic activation are closely intertwined (Tawakol et al., 2015). Conversely, glycolytic inhibition of macrophages using siRNA against PFKFB3 or using 3-[3-pyridinyl]-1-[4-pyridinyl]-2-propen-1-one, a selective inhibitor of PFKFB3, leads to a blockade in macrophage activation (Tawakol et al., 2015). *In vivo*, this translated into decreased ^18^F-fluorodexyglucose (^18^F-FDG) uptake in the arterial vessel wall in *Apoe*^–/–^ mice measured with positron emission tomography/computed tomography (Singh et al., 2016), underscoring attenuated arterial wall inflammation following attenuation of inducible glycolysis in macrophages (Tarkin et al., 2014). In our *Ldlr* knockout mice, we show that the progression of atherosclerosis is attenuated upon PFKFB3 inhibition. Plaques from mice treated with PFK158 displayed less fibrous cap atheromas and increased initial- and intermediate plaques, indicative of reduced formation of advanced plaques (Libby, 2012; Tomas et al., 2018). Thus, PFK158-treated mice displayed an increase in plaque stability index and thickening of the fibrous cap, which can be attributed to enhanced SMC proliferation and/or migration toward the fibrous cap (Allahverdian et al., 2018).

In mice, we showed that targeting PFKFB3, mainly expressed by macrophages and endothelial cell, *in vivo* led to decreased necrotic core area, a phenomenon that could be attributed to the significant loss of apoptotic cells in the PFK158-treated mice compared to the control group. In advanced stages of atherosclerotic plaques, macrophage apoptosis contributes to necrotic core formation, furthermore implying that glycolytic inhibition leads to plaque stabilization (Tabas, 2010; Libby, 2012; Tabas and Bornfeldt, 2016; Gonzalez and Trigatti, 2017).

As plaque progression is dependent on the influx of immune cells, we have investigated the metabolic state of PBMCs. The PBMC fraction comprises monocytes, which are key orchestrators in the development of atherosclerosis (Sotiriou et al., 2006; van der Laan et al., 2014; Nahrendorf and Swirski, 2016; Schnitzler et al., 2019). In patients with severe symptomatic coronary atherosclerosis, monocytes display a distinct glycolytic phenotype by overexpressing hexokinase 2 and PFKFB3 (Bekkering et al., 2016). We corroborated these findings as partial inhibition of PFKFB3 decreased glycolytic flux in PBMCs indicating that these cells were metabolically inhibited which in turn alleviated atherosclerotic burden in mice. However, as PBMCs include a variety of cells, such as monocytes and lymphocytes, it would be interesting to investigate which cell subset plays a predominant role in our model (Kleiveland, 2015).

The other pivotal cell type, the endothelial cells covering the atherosclerotic plaques, did not seem to be affected by glycolytic inhibition as ICAM-1, VCAM-1, and GLUT1 expression did not alter after treatment. This might be a result of treating the mice in this study with a low dose of PFK158, whereas *in vitro* studies used increased concentrations (Schnitzler et al., 2020). Concomitantly, we found increased plaque stability and thickening of the fibrous cap which may be consequences of either a decrease in SMC apoptosis or increased SMC proliferation. Loss of these cells contributes to plaque destabilization once exposed to increased endoplasmic reticulum stress which facilitates apoptosis of SMCs (Myoishi et al., 2007).

In summary, this study demonstrates that intraplaque PFKFB3 levels are associated with plaque stability and glycolytic inhibition leads to a decrease in vulnerable plaque characteristics and increases plaque stabilization. The present study identifies PFKFB3 inhibition as a novel target potentially suitable to further attenuate atherosclerotic development.

## Data Availability Statement

The raw data supporting the conclusions of this article will be made available by the authors, without undue reservation.

## Ethics Statement

The animal study was reviewed and approved by the Committee for Animal Welfare of the University of Amsterdam, Netherlands (protocol 265-AW-1), and comply to the European Regulations as identified in Directive 2010/63/EU on the protection of laboratory animals.

## Author Contributions

JS, KP, TS, and JK conceived and planned the experiments. JS, KP, A-MP, TS, and JK carried out the experiments and data analysis. JS, KP, TS, and JK wrote the manuscript. All authors provided critical feedback and helped shape the research, analysis and manuscript.

## Conflict of Interest

The authors declare that the research was conducted in the absence of any commercial or financial relationships that could be construed as a potential conflict of interest.
