# Supplementary material for: Inhibition of PFKFB3 Hampers the Progression of Atherosclerosis and Promotes Plaque Stability
Source: Front Cell Dev Biol. 2020 Nov 12;8:581641. doi: 10.3389/fcell.2020.581641 (PMC7688893; doi:10.3389/fcell.2020.581641)
Supplement: Supplementary file 1 [file Data_Sheet_1.docx]

***Supplementary material***

**
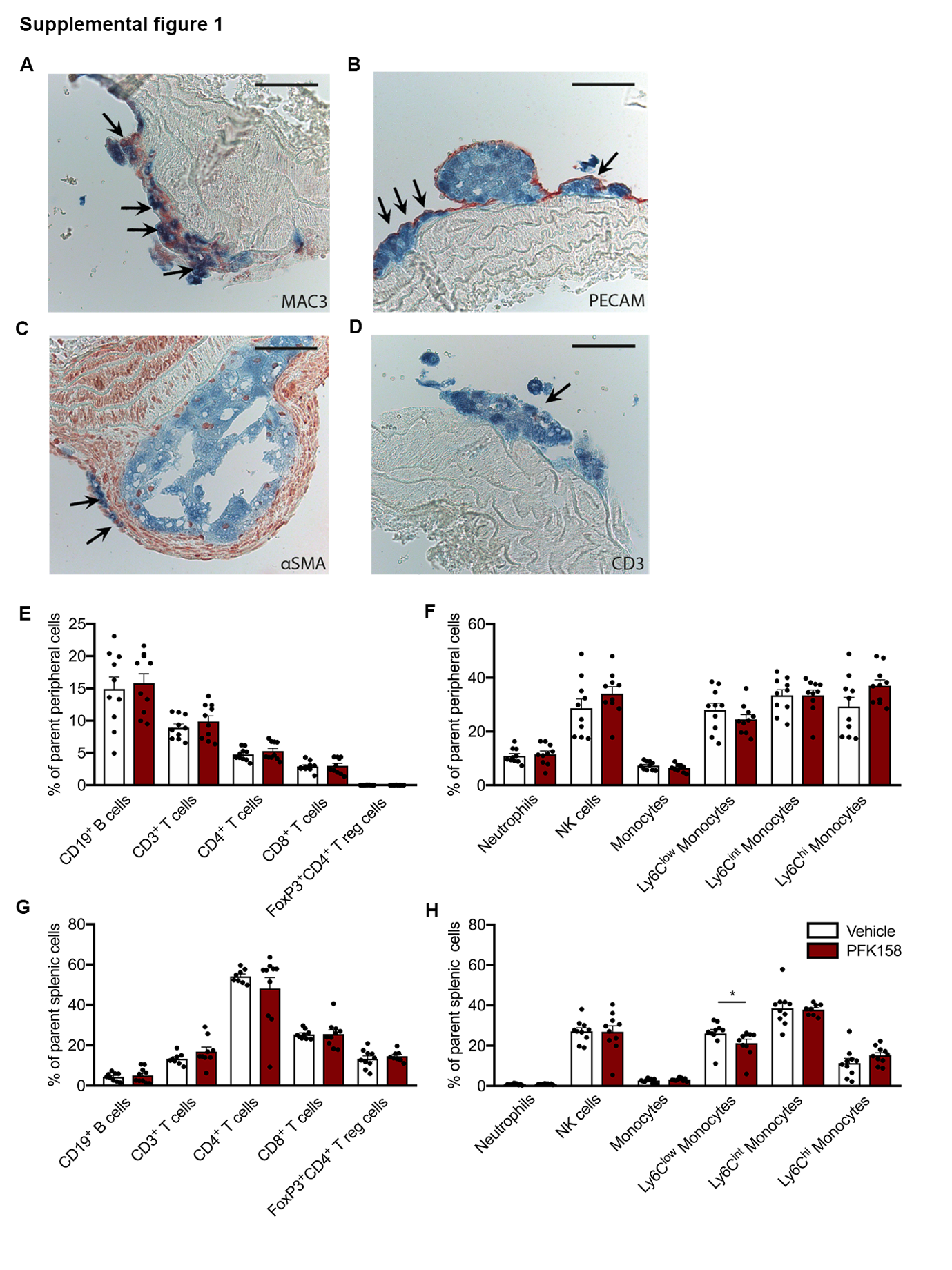
**

**Supplemental figure 1. PFKFB3 mostly present in macrophages and endothelium PFK158 treatment does not affect peripheral and splenic cell count.** (A) Immunohistochemistry of PFKFB3^+^ co-staining (blue) with MAC3^+^ macrophages (red); Scale bar 25μm, (B) PECAM^+^ endothelial cells (red); Scale bar 25μm, (C) α smooth muscle actin (αSMA)^+^ vascular smooth muscle cells (red); Scale bar 25μm and (D) CD3^+^ T cells (red); Scale bar 25μm.

(E) Peripheral lymphoid (n=10/group) and (F) peripheral myeloid cell subsets do not differ between groups (n=10/group) (G) Splenic lymphoid cells remain unaffected (n=10/group). (H) No changes in splenic myeloid subsets, only Ly6C^low^ monocytes decreased after PFK158 treatment (n=10/group). All test were performed using Two-tailed unpaired Mann-Whitney, P=0.0156 for Ly6C^low^ monocytes. Data are shown as mean ± standard error of the mean. *P<0.05. PFKFB3, 6-phosphofructo-2-kinase/fructose-2,6-biphosphatase 3; PECAM, platelet endothelial cell adhesion molecule; αSMA, α smooth muscle actin

**
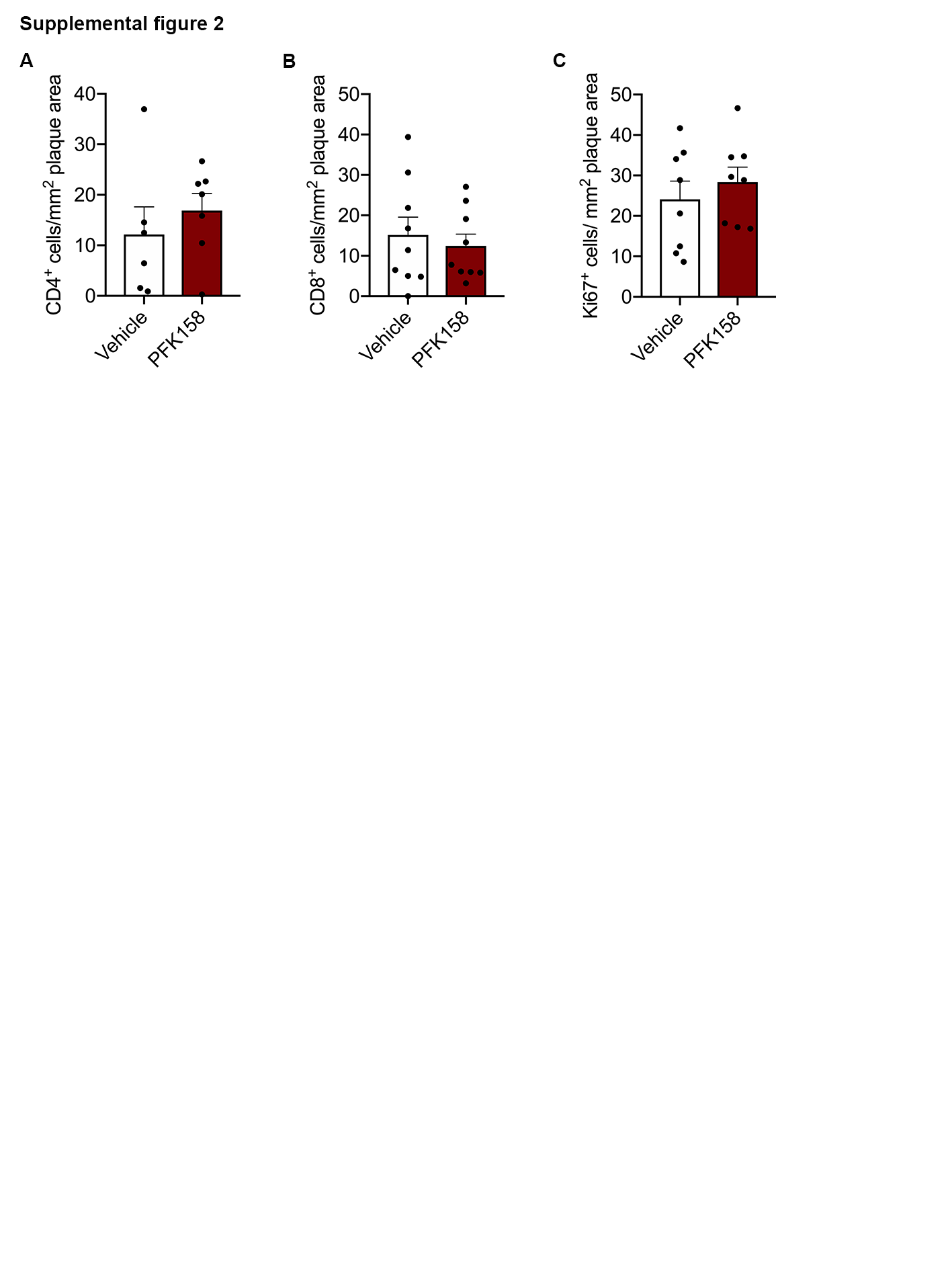
**

**Supplemental figure 2. No differences in lymphoid and proliferating cells were observed in the plaque. (**A) The number of CD4^+^ T cells (n=6 for vehicle; n=7 for PFK158), (B) CD8^+^ T cells (n=9/group) and (C) proliferating Ki67^+^ cells in the plaques do not alter after treatment with PFK158 (n=8) compared to vehicle (n=8). All test were performed using Two-tailed unpaired Mann-Whitney. Data are shown as mean ± standard error of the mean.

**
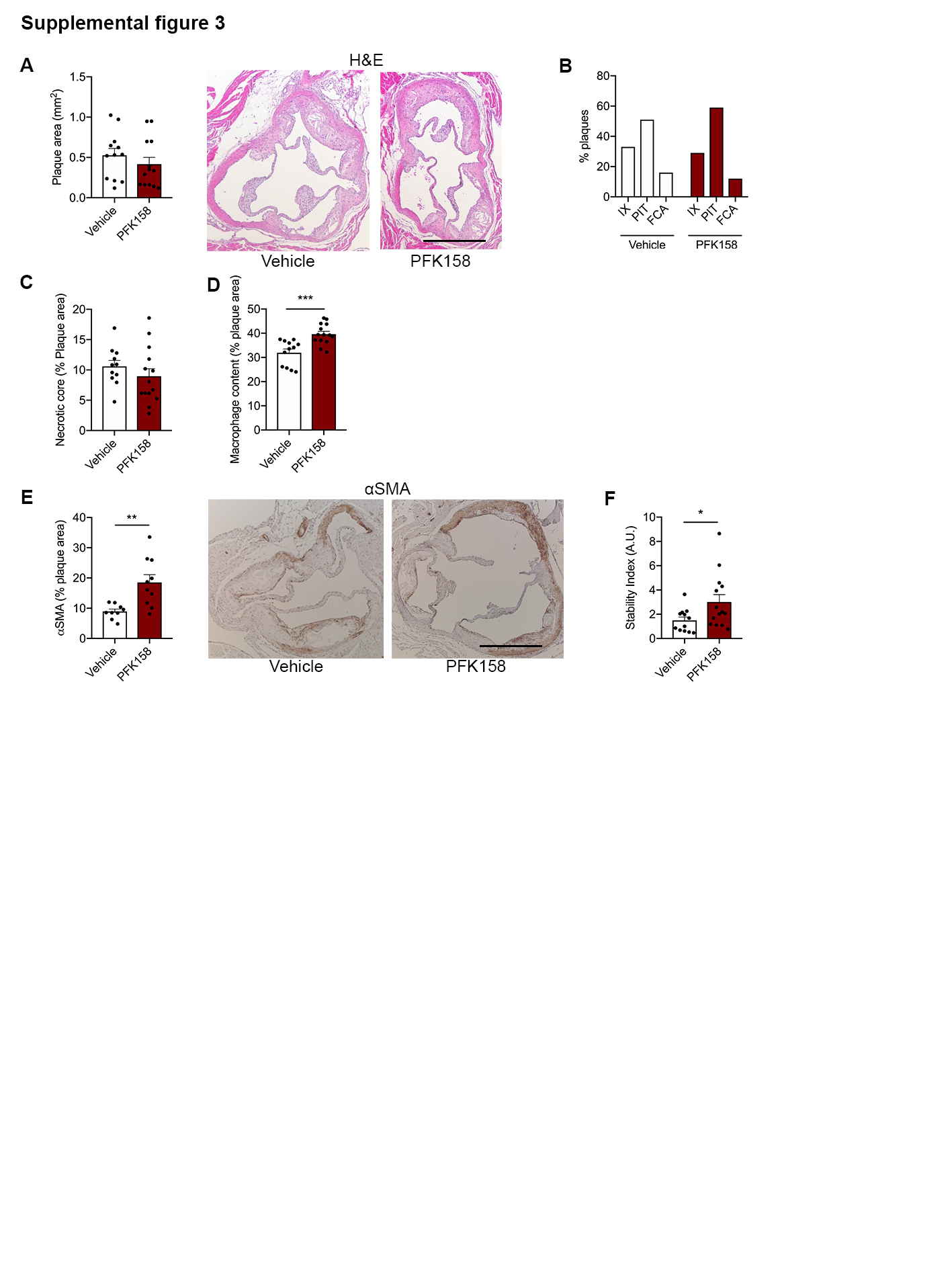
**

**Supplemental figure 3. PFKFB3 treatment increases plaque stability in aortic roots. (**A) Atherosclerotic lesion area in the aortic roots was similar between groups (n=12 vehicle vs n=13 PFK158; scale bar = 500 μm (B) Morphological analysis displayed no significant differences. (C) Necrotic core area was not affected by PFK158 treatment (n=11 vehicle vs n=14 PFK158). (D) Macrophage content increased in PFK158 treated mice (n=12 vehicle vs n=14 PFK158). Two-tailed unpaired Student T-test P=0.0004. (E) PFK158 induced a trend towards increased αSMA content (n=9 vehicle vs n=10 PFK158. Two-tailed unpaired Student T-test P=0.051; black bar = 500 μm. (F) Plaque stability index showed increased the plaque stability in PFK158 treated mice (n=13 vehicle vs n=14 PFK158. Two-tailed unpaired Mann-Whitney, P=0.027.

Data are shown as Mean ± standard error of the mean. *P<0.05, **P<0.005, *** P<0.0005 H&E Hematoxylin & eosin; IX initial xanthoma; PIT pathologic intimal thickening; FCA fibrous cap atheroma; αSMA, α smooth muscle actin

**Supplemental table 1. Primer sequences**

| **Primer** | **Sequence** |
| --- | --- |
| *Il1beta* | Fw: GCAACTGTTCCTGAACTCAACT  Rv: ATCTTTTGGGGTCCGTCAACT |
| *Cd36* | Fw: TGGCTAAATGAGACTGGGACC  Rv: GGCCATCTCTACCATGCCAA |
| *F4/80* | Fw: TGACAACCAGACGGCTTGTG  Rv: GCAGGCGAGGAAAAGATAGTGT |
| *Cd11c* | Fw: CGTCAGTACAAGGAGATGTTGGA  Rv: TCCTATTGCAGAATGCTTCTTTACC |
| *Cpt1a* | Fw: AGAGGGGAGGACAGAGACTG  Rv: TCTGCTCTGCCGTTGTTGTG |
| *Slc2a1* | Fw: TCGGGTATCAATGCTGTGTTCT  Rv: CCGTGTTGACGATACCGGAG |
| *Slc2a3* | Fw: ACCAAGTGAGGGACTGCTGA  Rv: CATAGAGTTGCGTCTGCCAAA |
| *Pfkfb3* | Fw: ATGCTGGTGTGTGTGAGGAA  Rv: GATCCTGGTAGGACTCTCCCG |
| *Cs* | Fw: GCAGCAGTATCGGAGCCATT  Rv: CACCCTCATGGTCACTATGGAT |
| *Fh1* | Fw: AATAAAAGCCGCCATGCCAA  Rv: GTGACAAAAGGCAAACCTGTGA |
